# The Role of Hippo Signaling in Brain Arteriovenous Malformations: Molecular Insights into Post-Embolization Remodeling

**DOI:** 10.3390/ijms26083791

**Published:** 2025-04-17

**Authors:** Belal Neyazi, Vanessa Magdalena Swiatek, Mohammad Ali Karimpour, Sarah Stassen, Klaus-Peter Stein, Ali Rashidi, Claudia Alexandra Dumitru, I. Erol Sandalcioglu

**Affiliations:** Department of Neurosurgery, Otto-von-Guericke University, 39120 Magdeburg, Germany; vanessa.swiatek@med.ovgu.de (V.M.S.); mohammad.karimpour@uk-koeln.de (M.A.K.);

**Keywords:** brain arteriovenous malformation, embolization, Yes-associated protein, connective tissue growth factor, cysteine-rich angiogenic inducer 61

## Abstract

Brain arteriovenous malformations (bAVMs) are complex vascular lesions with significant clinical risks. The Hippo signaling pathway, particularly its downstream effector YAP, plays a crucial role in angiogenesis and vascular remodeling. This study investigates the role of YAP and related molecular markers in bAVMs, focusing on the effects of embolization. Immunohistochemical analysis was conducted on tissue samples from bAVM patients (n = 127), as well as on healthy blood vessels (n = 17). YAP, HIF-1α, FGFR1, CTGF, and CYR61 expression were quantified and correlated with clinical parameters. Results: In healthy vessels, YAP exhibited nuclear localization in (sub)endothelial cells and the tunica media, while CTGF and CYR61 were detected in the cytoplasm and extracellular matrix. The expression of YAP, CTGF, and CYR61 was significantly lower in bAVM tissues. Embolized bAVMs exhibited significantly higher expression of YAP, CTGF, and CYR61 compared to non-embolized tissues, suggesting a link between embolization and pro-angiogenic signaling. Additionally, FGFR1 was upregulated in embolized tissues. These results suggest that upregulation of YAP expression via the Hippo pathway might play a key role in bAVM pathophysiology. Embolization may further promote vascular remodeling. Dysregulation of YAP and related molecules in bAVMs warrants further studies to explore potential therapeutic strategies targeting the Hippo pathway.

## 1. Introduction

Brain arteriovenous malformations (bAVMs) are complex vascular anomalies characterized by direct arteriovenous shunting without an intervening capillary network, leading to high-flow dynamics and potential hemodynamic instability [1,2]. The incidence of bAVMs ranges between 0.89 and 1.34 per 100,000 patient years, with the most common clinical manifestation being intracerebral hemorrhage due to rupture of the nidus. The mortality rate after the first hemorrhage exceeds 50% [3], emphasizing the critical need to better understand the underlying molecular mechanisms of bAVM pathophysiology.

Among the various signaling pathways that regulate vascular development and stability, the Hippo signaling pathway has recently gained attention for its role in endothelial cell behavior, mechanotransduction, and vascular remodeling [4,5]. The pathway’s core effector, Yes-associated protein (YAP), is a transcriptional coactivator that regulates genes essential for angiogenesis, extracellular matrix remodeling, and cell proliferation [4,6]. In its inactive state, the Hippo signaling pathway is suppressed by various external stimuli, including hypoxia, growth factors, turbulent blood flow, and increased extracellular matrix (ECM) stiffness. This suppression prevents the phosphorylation cascade of various mediators, thereby maintaining the pathway in an inactive state. Consequently, the Yes-associated protein (YAP) and its paralog transcriptional coactivator transcription regulator 1 (TAZ) remain dephosphorylated, allowing their nuclear translocation, where they bind to TEA domain family member transcription factors. This interaction induces the transcriptional activation of key target genes, including connective tissue growth factor (CTGF) and cysteine-rich angiogenic inducer 61 (CYR61), which play central roles in ECM remodeling, angiogenesis, and vascular proliferation as well as regulating endothelial proliferation, migration, and survival [4]. Their activation is influenced by various cellular cues, including mechanical stress, cell adhesion, and inflammatory signals, all of which are relevant in vascular remodeling processes [5]. Previous studies have demonstrated increased YAP activity in pathological angiogenesis, such as in age-related macular degeneration and tumor neovascularization, suggesting a pivotal role in aberrant vascular structures [6,7,8].

Despite its recognized function in angiogenesis and mechanotransduction, the Hippo pathway has not been systematically investigated in the context of bAVMs. However, several key features of bAVMs—such as the absence of a capillary bed, disturbed flow patterns, ECM disorganization, and increased tissue stiffness—represent strong biophysical and biochemical stimuli for Hippo pathway modulation [9,10,11]. In particular, YAP/TAZ are known to respond to shear stress and ECM cues, both of which are profoundly altered in bAVMs [9,10,12]. Moreover, recent vascular studies have implicated Hippo signaling in endothelial plasticity and vascular malformations [11,13], making it a compelling candidate pathway to explore in bAVM tissue.

Two of the major YAP target genes, CTGF and CYR61, are extracellular matrix-associated signaling molecules that regulate endothelial cell function, angiogenesis, and tissue remodeling [14]. Both genes are transcriptionally activated in response to proangiogenic signals such as vascular endothelial growth factor (VEGF) and fibroblast growth factor (FGF) [14,15], and their dysregulation has been implicated in tumor angiogenesis and vascular pathology. In mice, genetic deletion of CTGF or CYR61 leads to severe vascular defects, highlighting their essential role in vascular stability [14]. Despite their well-documented involvement in vascular biology, their role in bAVM pathophysiology remains undefined.

Based on these considerations, we hypothesized that dysregulation of the Hippo pathway—particularly through altered YAP activity—may contribute to the pathological vascular remodeling observed in bAVMs. In this study, we present the first systematic analysis of YAP expression in bAVM vessels compared to control vessels. Additionally, we investigate the expression patterns of CTGF, CYR61, hypoxia-inducible factor 1-α (HIF-1α), and FGFR1 in bAVM tissue and analyze their correlation with relevant clinical parameters. Our findings provide novel insights into the molecular mechanisms underlying bAVM and may contribute to identifying potential therapeutic targets in the treatment of these challenging lesions.

## 2. Results

### 2.1. Expression Patterns of HIF-1α, FGFR1, YAP, CTGF, and CYR61 in bAVM

Immunohistochemical analysis of HIF-1α, FGFR1, YAP, CTGF, and CYR61 revealed distinct expression patterns in healthy vessels (control specimens). HIF-1α and YAP exhibited a nuclear expression pattern in (sub)endothelial cells as well as in the tunica media. FGFR1 showed membrane-associated positive signals, with predominant expression in endothelial cells. CTGF and CYR61 were detected both in the cytoplasm and as components of the extracellular matrix.

**Figure 1:**
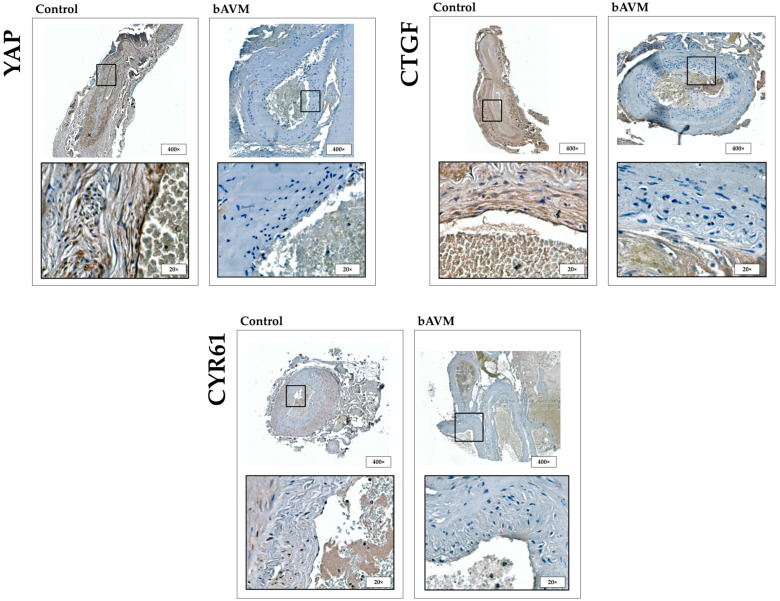
YAP expression in endothelial cells (circle), subendothelial, and perivascular tissue in control vessels. Absence of YAP expression in bAVM vessels. CTGF expression in endothelial cells (circle), subendothelial, and perivascular tissue in control vessels. Absence of CTGF expression in bAVM vessels. CYR61 expression in endothelial cells (circle), subendothelial, and perivascular tissue in control vessels. Absence of CYR61 expression in bAVM vessels. Higher-resolution, zoomed-in views of lower panels—highlighting subcellular localization patterns (e.g., nuclear vs. cytoplasmic staining)—are provided in the Supplementary Material.

**Figure 2:**
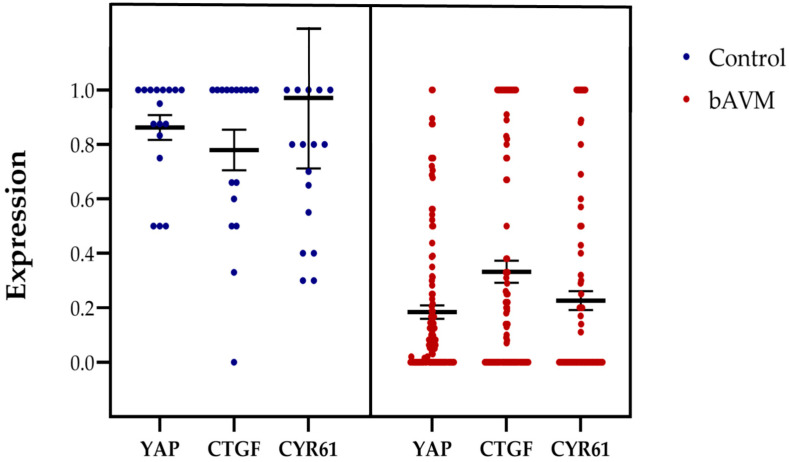
Representation of the average expression levels of YAP, CTGF, and CYR61 in control tissue compared to bAVM tissue. YAP scoring was performed using a 5-tier scale, where a score of 4 corresponds to 1 (100%) in this bar chart. For CTGF and CYR61, a score of 1 indicates positive expression of the marker in the ECM of the tunica media, representing a proportional value of 100%. The control group consisted of 17 samples, while the bAVM group included 127 samples. Error bars represent the standard error of the mean, and the thick bars indicate the mean values.

In comparison, bAVM tissue exhibited a significantly lower expression of YAP in (sub)endothelial cells and the tunica media (*p* < 0.001), as well as reduced expression of CTGF (*p* < 0.001) and CYR61 (*p* < 0.001) in the cytoplasm and ECM (Figure 1 and Figure 2).

### 2.2. Correlation Between YAP, CTGF, and CYR61 Expression and Clinical Parameters

Antigen expression levels showed no significant differences concerning patient-related factors such as sex, age at surgery, arterial hypertension, or smoking status. Additionally, no correlation was found between antigen expression and rupture status or symptoms such as epilepsy and focal neurological deficits. The only significant association with clinical symptoms was an increased expression of HIF-1α in patients experiencing headaches (*p* = 0.042). Patients with a lower preoperative modified Rankin Scale (mRS) score exhibited significantly higher expression levels of CTGF (*p* = 0.031; rho = −0.228), CYR61 (*p* = 0.011; rho = −0.256), and HIF-1α (*p* = 0.047; rho = −0.210) (Table 1).

Expression levels also varied by anatomical location, with significantly higher YAP (*p* = 0.006) and CYR61 (*p* = 0.046) levels in supratentorial compared to infratentorial regions. Additionally, increased HIF-1α concentrations were significantly associated with eloquent brain regions. A positive correlation was observed between AVM size and the expression of YAP (*p* = 0.015; rho = 0.252), CTGF (*p* = 0.019; rho = 0.249), CYR61 (*p* = 0.031; rho = 0.227), and HIF-1α (*p* = 0.004; rho = 0.311), whereas none of the biomarkers correlated with Spetzler–Martin grade (Table 1).

### 2.3. Embolization Induced Expression of YAP, CTGF and CYR61 Mediated by HIF-1α and FGFR1

Patients who underwent preoperative embolization (n = 22) exhibited higher expression levels of YAP, CTGF, and CYR61, among other markers, compared to those who did not receive preoperative therapy (n = 97) (Table 1). Notably, HIF-1α and FGFR1, which are considered potential inducers of the Hippo signaling pathway and regulators of YAP, CTGF, and CYR61 expression, were also found to be upregulated.

**Figure 3:**
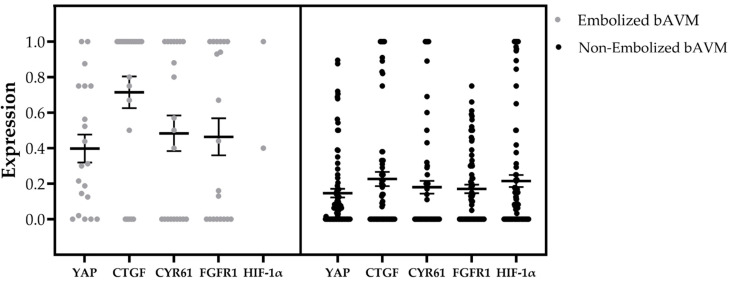
Representation of the average expression levels of the antigens YAP, CTGF, CYR61, FGFR1, and HIF-1α, comparing preoperatively embolized and non-embolized tissue samples. YAP and HIF-1α were scored using a 5-tier scale, where a score of 4 corresponds to 1 (100%) in this bar chart. For CYR61, CTGF, and FGFR1, a score of 1 indicates positive expression in the ECM of the tunica media or in the cytoplasm of (sub)endothelial cells, representing a proportional value of 100%. The embolized group consisted of 22 samples, while the non-embolized group included 97 samples. Error bars represent the standard error of the mean, and the thick bars indicate the mean values.

**Figure 4:**
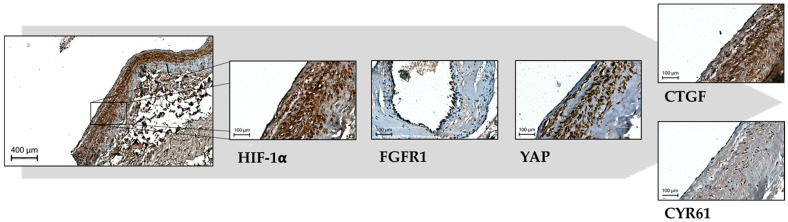
Immunohistochemical representation of HIF-1α, FGFR1, YAP, CTGF, and CYR61 in embolized bAVM tissue. The first image is acquired at 100× magnification, all other images at 400× magnification. This figure serves as a schematic overview to illustrate the proposed relationship between YAP expression and its downstream targets CTGF and CYR61, potentially mediated by upstream regulators HIF-1α and FGFR1.

Given this, we investigated whether the induction of HIF-1α and FGFR1 was more pronounced in preembolized bAVM tissue compared to non-embolized bAVM tissue, as suggested by the initial findings. The analysis confirmed a significant upregulation of both HIF-1α (*p* ≤ 0.001) and FGFR1 (*p* = 0.035) in preembolized bAVM tissue compared to the non-embolized cohort (Table 1; Figure 3 and Figure 4).

Further analysis of the individual parameters revealed a strong correlation between HIF-1α levels and the expression of YAP (*p* ≤ 0.001, rho = 0.689), CTGF (*p* ≤ 0.001, rho = 0.740), and CYR61 (*p* ≤ 0.001, rho = 0.646) in bAVM patients, suggesting that HIF-1α may serve as an inducer of YAP, CTGF, and CYR61. Additionally, a correlation was observed between FGFR1 expression and the expression of YAP (*p* ≤ 0.001, rho = 0.437), CTGF (*p* = 0.005, rho = 0.287), and CYR61 (*p* = 0.08, rho = 0.261).

When analyzing preembolized patients separately, the significant association between HIF-1α and components of the Hippo signaling pathway was particularly evident for CTGF (*p* ≤ 0.001, rho = 0.718) and CYR61 (*p* = 0.027, rho = 0.483). For YAP, a clear trend toward an association with HIF-1α expression was observed (*p* = 0.058, rho = 0.431).

## 3. Discussion

The molecular pathology of bAVMs remains insufficiently understood, and current treatment criteria primarily focus on intervention-associated risks while neglecting long-term disease-related factors. A more comprehensive risk stratification approach that integrates morphological imaging assessments with biological markers could improve patient outcomes and guide more personalized therapeutic decisions. This study highlights the Hippo signaling pathway as a key regulator of bAVM pathophysiology, demonstrating its role in disease progression and treatment response. Our findings suggest that interventional procedures influence cellular signaling, potentially exacerbating therapy-associated risks.

The Hippo pathway plays a fundamental role in vascular homeostasis, angiogenesis, and mechanotransduction. Under normal conditions, external signals such as hypoxia, growth factors, turbulent blood flow, and a stiff ECM suppress Hippo pathway activation. This inhibition prevents the phosphorylation of various mediators, thereby maintaining the pathway in an inactive state. Consequently, YAP/TAZ remains dephosphorylated and translocates into the nucleus, where they interact with TEA domain family member 1, driving the transcription of pro-angiogenic and ECM-remodeling genes such as CTGF and CYR61 [16]. These downstream factors promote vascular remodeling, angiogenesis, and pathological vessel proliferation, which may contribute to bAVM progression.

The altered expression patterns of YAP, CTGF, and CYR61 in bAVM suggest a potential dysregulation of the Hippo signaling pathway. In healthy vessels, YAP exhibited nuclear localization in (sub)endothelial cells and the tunica media, indicating active signaling through the Hippo pathway. Similarly, CTGF and CYR61 were found in both the cytoplasm and extracellular matrix, suggesting their involvement in vascular remodeling and endothelial function. However, the results in bAVM tissue were not what we initially expected. In contrast to retinal diseases and certain cancers, where the Hippo pathway is usually off and YAP is upregulated [17], we anticipated a similar upregulation of YAP in bAVMs. Instead, bAVM tissue displayed significantly lower expression of YAP in (sub)endothelial cells and the tunica media, as well as reduced expression of CTGF and CYR61 in the cytoplasm and extracellular matrix. This unexpected finding leads us to hypothesize that the Hippo pathway might be dysregulated in bAVMs, but the underlying mechanisms could be different from what is seen in retinal-related diseases and cancer. YAP and TAZ are well-established mechanosensors that respond to shear forces, cell-cell interactions, and ECM stiffness [9,10]. In bAVMs, characterized by direct arteriovenous shunting without an intervening capillary network, the altered hemodynamic environment may impair YAP/TAZ activation, leading to reduced expression of their target genes. Given that CYR61 and CTGF play essential roles in ECM remodeling and endothelial cell migration [18], their downregulation could contribute to increased vascular fragility, a hallmark of bAVMs, and may increase the risk of rupture.

Although VEGF is one of the most extensively studied angiogenic factors in bAVM pathophysiology, it was not included in our panel of investigated markers. Its upregulation in bAVM has already been well documented in multiple studies, including work from our own group, which demonstrated significantly elevated VEGF plasma levels in bAVM patients and strong VEGF immunoreactivity in resected specimens [19,20]. Given this established role, the current study focused instead on exploring less-characterized molecular mechanisms—specifically the Hippo signaling pathway and its downstream effectors YAP, CTGF, and CYR61. While VEGF does not constitute a core component of the Hippo cascade, it can influence Hippo pathway activity indirectly via upstream mediators such as HIF-1α and FGFR1. Hypoxia-induced stabilization of HIF-1α, a well-known driver of VEGF expression, has been shown to repress Hippo kinases and thereby promote nuclear YAP translocation [21]. Similarly, FGFR1 activation has been linked to YAP activation through reciprocal regulatory loops [22]. Nevertheless, the canonical Hippo pathway (MST1/2–LATS1/2–YAP/TAZ) operates independently of VEGF signaling per se [23]. Therefore, the current analysis was designed to remain focused on the intrinsic regulation and dysregulation of the Hippo–YAP axis in bAVMs, without duplicating previously established findings regarding VEGF.

In further analyses, we found that YAP and HIF-1α were predominantly localized to the nucleus in both preoperatively embolized and non-embolized bAVM tissues. This supports the hypothesis that these molecular regulators are key drivers of angiogenesis in bAVMs. Previous studies linked YAP nuclear translocation to mechanical compression, hypoxia, and conditions involving increased matrix stiffness or mechanical tension, which promote Hippo pathway inactivation and vascular remodeling [9,10,12,24]. In contrast, cells cultured on softer substrates without mechanical stress showed YAP localization in the cytoplasm, suggesting Hippo pathway activation and the suppression of angiogenesis [9,10]. In bAVM vessels, increased stiffness, likely due to disturbed collagen expression, may contribute to the nuclear YAP translocation [25,26]. Additionally, oscillatory shear stress, a known contributor to vascular malformations, has been shown to promote pro-angiogenic signaling [11]. Our results indicate that higher nuclear YAP localization in embolized bAVMs might be associated with aberrant vascular remodeling, further driving pathological vessel growth compared to non-embolized bAVMs. This process becomes particularly significant after embolization, where mechanical changes in ECM stiffness could trigger YAP activation, leading to enhanced vascular proliferation and remodeling.

A further consequence of reduced YAP/TAZ activity may be impaired arterial differentiation. The interaction of YAP/TAZ with the NOTCH signaling pathway and the regulation of Delta-like 4 expression are crucial for maintaining arterial identity [13]. Decreased YAP/TAZ expression in bAVMs could therefore exacerbate incomplete endothelial differentiation and reinforce the aberrant arteriovenous structure. Additionally, the absence of increased HIF-1α and FGFR1 expression in non-embolized bAVMs suggests that hypoxia-driven angiogenic signaling is not a dominant feature prior to embolization.

In addition to ECM-related mechanical cues, hemodynamic changes following embolization may also contribute to YAP activation. bAVMs are characterized by high-flow, low-resistance vascular shunts, and embolization-induced occlusion increases nidus resistance, reduces laminar shear stress, and enhances turbulent flow. Previous studies have shown that turbulent blood flow promotes YAP nuclear translocation and the expression of CTGF and CYR61, key mediators of ECM remodeling [27]. Moreover, bAVM endothelial cells are particularly sensitive to oscillatory shear stress, which could amplify YAP activation following embolization [11].

Our findings are consistent with hemodynamic studies that show accelerated blood flow in residual bAVM shunts after partial embolization [28]. Elevated Reynolds numbers post-intervention indicate a shift from laminar to turbulent flow, a phenomenon associated with vascular remodeling and pathology in other vascular diseases [29]. Since turbulence-induced YAP activation has been linked to endothelial dysfunction and abnormal vessel growth [27,30], our results suggest that embolization may inadvertently exacerbate vascular instability through a YAP-dependent mechanotransduction pathway.

FGFR1, a transmembrane receptor closely linked to pro-angiogenic VEGF signaling, plays a significant yet underexplored role in bAVM pathophysiology [20,21]. Our analysis detected membrane-associated FGFR1 expression in bAVMs, and we observed a strong correlation between FGFR1 and YAP expression, suggesting a potential signaling interaction in bAVM remodeling. Previous studies have demonstrated that FGFR1 and YAP engage in a reciprocal amplification loop, where YAP enhances FGFR1 transcription, while FGFR1 promotes YAP activation via Hippo pathway suppression [30]. Additionally, FGFR1 binds to CTGF, further modulating ECM remodeling and vascular instability [31]. These interactions suggest that FGFR1-YAP crosstalk may represent a key molecular mechanism underlying embolization-induced vascular changes.

Our findings highlight the potential risks associated with embolization-induced YAP expression. While embolization remains a valuable treatment for bAVMs, its impact on molecular pathways that promote pathological vascular remodeling must be considered. The observed YAP-HIF-1α-FGFR1 interactions suggest that targeting YAP signaling could offer a novel therapeutic strategy to reduce post-embolization neovascularization and rupture risk.

This study is limited by its retrospective design, which restricted the completeness of clinical data. The small sample size and exclusion of non-surgically treated patients may limit generalizability, particularly for high-grade AVMs, which were underrepresented. The lack of in vivo validation raises questions about whether molecular pathways were altered post-resection. Additionally, the time interval between embolization and surgery was not accounted for, potentially affecting expression dynamics. Tissue preservation issues, antibody specificity, and subjective scoring introduce further variability, though efforts were made to minimize bias. Despite these limitations, our findings provide valuable molecular insights into bAVM pathophysiology, warranting further prospective studies.

## 4. Materials and Methods

### 4.1. Tissue Collection and Patient Data

**Figure 5:**
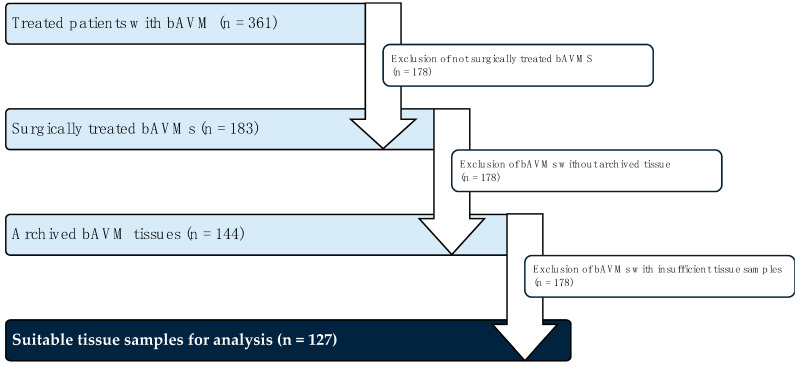
Flowchart depicting the cohort selection process, reasons for exclusion, and the number of excluded bAVM tissue samples and patients.

Between 1995 and 2019, a total of 361 patients with bAVMs underwent treatment at the Departments of Neurosurgery at Nordstadt Hospital Hanover and University Hospital Magdeburg. For this study, we retrospectively analyzed a subset of 127 patients who underwent surgical treatment, evaluating both clinical parameters and formalin-fixed paraffin-embedded (FFPE) bAVM tissue samples. The diagnosis of an AVM was previously confirmed by a pathologist using a hematoxylin–eosin (H&E) overview staining. A corresponding histopathological report is available for each case. The cohort selection process and reasons for exclusion are illustrated in Figure 5. The study cohort included patients aged 11 to 90 years (mean age: 45 years). A summary of patient characteristics is provided in Table 2.

This study was approved by the Ethics Committee of Hanover Medical School and the Medical Faculty of Otto von Guericke University (NOVA Nr. 68/20 and RENOVA Nr. 94/20), which also granted a waiver for informed written consent.

### 4.2. Immunhistochemistry

#### 4.2.1. Control Specimen

Seventeen cerebral control specimens were analyzed. Six samples of the arteria meningea media were obtained from patients undergoing emergency craniotomy, while eleven arteria temporalis biopsy samples were collected from patients initially suspected of arteritis but without histopathological evidence of inflammation. Negative controls were processed by omitting the primary antibody. Paraffin-embedded glioblastoma specimens served as positive controls for all investigated antibodies.

#### 4.2.2. TMA: Construction and Immunohistochemistry

To identify the region of interest within bAVM specimens, H&E staining was performed. Tissue cores were extracted from FFPR bAVM specimens using a 3 mm biopsy punch and transferred into recipient blocks. Sections (2 μm) were cut, deparaffinized, and subjected to heat-induced epitope retrieval in citrate buffer (pH 6.0).

Immunohistochemical staining was performed using the following primary antibodies: anti-YAP (1:250; anti-human YAP F(ab′)2 mouse monoclonal antibody, Santa Cruz Biotechnology, Santa Cruz, CA, USA), anti-CTGF (1:50; anti-CTGF 6B13 mouse monoclonal antibody, Santa Cruz Biotechnology, Santa Cruz, USA), anti-CYR61 (1:50; anti-CYR61 A-10 mouse monoclonal antibody, Santa Cruz Biotechnology, Santa Cruz, USA), anti-HIF-1α (1:1000; polyclonal rabbit antibody, Proteintech, USA), and anti-FGFR1 (FGF Receptor 1 (D8E4) XP, rabbit monoclonal antibody, Cell Signaling Technology, Danvers, MA, USA). Secondary and colorimetric reactions were conducted using the UltraVision™ LP Detection System, following the manufacturer’s protocol (Thermo Scientific, Fremont, CA, USA). Counterstaining was performed with hematoxylin (Carl Roth, Karlsruhe, Germany), and sections were mounted using Mountex^®^ (Medite, Burgdorf, Germany).

Digital imaging of stained sections was performed using an Aperio VERSA whole-slide scanner at high resolution. Image analysis was conducted using Aperio ImageScope software version 12.3 (Leica Biosystems, Nussloch, Germany).

#### 4.2.3. Histological Analysis

Nuclear immunostaining of the (sub)endothelium and perivascular space was documented for YAP and HIF-1α, while cytoplasmic expression in the (sub)endothelium was analyzed for CTGF, CYR61, and FGFR1.

To quantify nuclear YAP and HIF-1α expression, a modified 5-tier scoring system was applied:

- Score 0: <20% positive nuclear expression.
- Score 1: 20–40% positive nuclear expression.
- Score 2: 40–60% positive nuclear expression.
- Score 3: 60–80% positive nuclear expression.
- Score 4: 80–100% positive nuclear expression.

**Figure 6:**
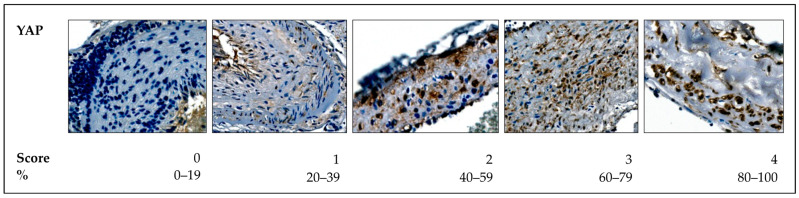
Modified 5-tier score using the example of the nuclear expression of YAP.

A score of 0 was classified as low expression, while a score of 4 indicated strong expression. The scores for all vessels in each sample were averaged to determine the mean positive expression per specimen. Samples with a score of ≥1 were considered positive for (sub)endothelial expression (Figure 6).

**Figure 7:**
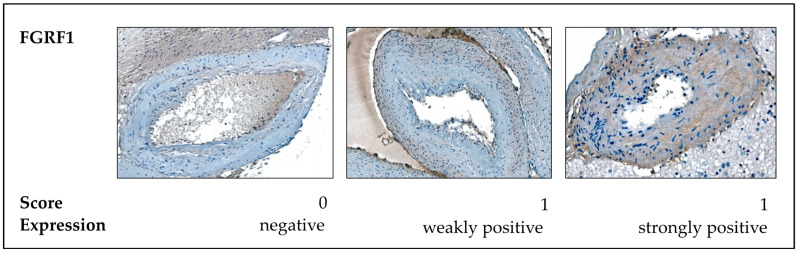
Two-tier score using the example of the cytoplasmic expression of FGFR1.

For CTGF, CYR61, and FGFR1, a cytoplasmic expression pattern was observed in the (sub)endothelium. To assess their expression, a binary (2-tier) scoring system was applied, classifying vessels as either positive (1 point) or negative (0 points) based on staining intensity (Figure 7).

**Figure 8:**
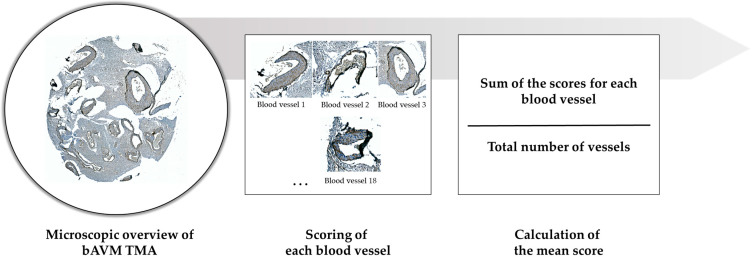
Schematic illustration of the scoring principle. First, a microscopic overview of the entire TMA (shown here for a bAVM sample) is provided. Depending on the protein analyzed, each vessel was evaluated using either a modified 5-tier or a 2-tier scale. Individual scores were then summed and divided by the number of vessels to calculate the mean score.

Quantitative analysis was performed for each individual vessel identified on the TMA core of each patient sample. Every vessel was scored individually, and the total score per patient was calculated by summing all individual vessel scores and dividing by the total number of vessels analyzed (Figure 8). This approach yielded a mean expression score for each patient and enabled consistent and reproducible comparisons across the study cohort.

All slides were scanned using a high-resolution whole-slide scanner, and digital analysis was conducted using specialized imaging software capable of up to 400× magnification. Importantly, the entire vessel structure was assessed in each TMA core, rather than selected subregions or fields of view, minimizing sampling bias (Figure 8). While the ability to digitally zoom in was essential for the reliable identification of subcellular localization—particularly to distinguish nuclear from cytoplasmic staining—the magnification did not influence scoring outcomes, as all vessels were evaluated in full. All immunohistochemical scoring was performed independently by M.A.K., B.N., and S.S. to ensure consistency and reproducibility.

#### 4.2.4. Statistics

All statistical analyses were conducted using SPSS™ for Windows 18.0 (SPSS Inc., Chicago, IL, USA) and GraphPad Prism 5 (GraphPad Inc., San Diego, CA, USA). The Kolmogorov–Smirnov test was used to assess deviations from normal distribution. For correlation analyses, Pearson’s correlation test was applied for normally distributed data, while Spearman’s rank correlation (Spearman’s Rho) was used for non-normally distributed variables. Comparisons between groups were performed using the non-parametric Mann–Whitney U test. A *p*-value of ≤0.05 was considered statistically significant for all analyses.

## 5. Conclusions

This study proposes that the Hippo signaling pathway, particularly YAP expression, plays a significant role in the progression and post-embolization remodeling of bAVMs. We hypothesize that embolization-induced upregulated expression of YAP may contribute to pathological vascular remodeling and neovascularization. The interactions between YAP, HIF-1α, and FGFR1 could potentially serve as therapeutic targets to address post-embolization vascular instability. However, our findings regarding the altered expression patterns of YAP, CTGF, and CYR61 in bAVM tissue suggest a potential dysregulation of the Hippo pathway, which is different from what we initially expected based on findings in retinal diseases and certain cancers, where the Hippo pathway is typically inactive, and YAP is upregulated. In contrast, bAVM tissue showed significantly lower expression of YAP and reduced CTGF and CYR61 expression. This discrepancy points to the complexity of Hippo pathway regulation across different tissues and highlights the need for further investigation to fully understand its role in vascular malformations.

## Figures and Tables

**Table 1:** Association between the analyzed markers and various clinical parameters. Statistical analysis was performed with the Mann–Whitney U-test for categorical and Spearman’s rank for continuous variables. Values of *p* < 0.05 were considered statistically significant and are indicated by an asterisk (*) in the table.

Clinical Parameter	YAP (*p*-Value)	CTGF (*p*-Value)	CYR61 (*p*-Value)	HIF-1α (*p*-Value)	FGFR1 (*p*-Value)
Sex	0.386	0.557	0.173	0.786	0.134
Age	0.144	0.835	0.729	0.092	0.051
Hypertension	0.606	0.760	0.519	0.489	0.506
Nicotine abuse	0.872	0.774	0.676	0.671	0.842
Localization (supra-/infratentorial)	* 0.006	0.108	* 0.046	0.059	0.106
Rupture status	0.247	0.130	0.080	0.133	0.311
Spetzler–Martin grade	0.871	0.954	0.993	0.677	0.910
bAVM size	* 0.015	* 0.019	* 0.031	* 0.004	0.087
Eloquent localization	0.348	0.183	0.285	* 0.047	0.890
Headache	0.558	0.112	* 0.042	0.919	0.713
Epilepsy	0.732	0.956	0.921	0.981	0.891
Neurological deficits	0.945	0.895	0.695	0.823	0.923
Preoperative mRS	0.102	* 0.031	* 0.011	* 0.047	0.513
Preoperative embolization	* 0.001	* ≤0.001	* 0.009	* ≤0.001	* 0.035

**Table 2:** Clinical and demographic characteristics of the study cohort (n = 127), presenting absolute numbers and corresponding percentages for sex, localization, rupture status, associated intranidal aneurysms, bAVM size, Spetzler–Martin grading, comorbidities, symptoms, preoperative modified Rankin Scale, and prior treatment before surgical therapy.

	Total Number (n = 127)	Percentage (100%)
Sex		
Male	68	53.5
Female	59	46.5
Localization		
Supratentorial	103	81.1
Infratentorial	23	18.1
Missing information	1	0.8
Rupture status		
Unruptured	27	21.3
Ruptured	99	78.0
Missing information	1	0.7
Associated intranidal aneurysms		
Yes	55	43.3
No	16	12.6
Missing information	56	44.1
bAVM size		
<3 cm	73	57.5
3–6 cm	28	22.0
>6 cm	0	0
Missing information	26	20.5
Spetzler–Martin Grading		
Grade I	29	22.8
Grade II	26	20.5
Grade III	26	20.5
Grade IV	4	3.1
Missing information	42	33.1
Comorbidities		
Hypertension	42	33.1
Nicotine abuse	23	18.1
Symptoms		
Headache	47	37.0
Epilepsy	40	31.5
Neurological deficits	87	68.5
Preoperative mRS		
0	3	2.4
1	26	20.5
2	15	11.8
3	12	9.4
4	26	20.5
5	33	26.0
Missing information	12	9.4
Treatment prior to surgical therapy		
Embolization	22	17.3
Radiotherapy	2	1.6

## Data Availability

The datasets obtained and analyzed during the current study are available from the corresponding author upon reasonable request.

## Supplementary Materials

The following supporting information can be downloaded at: https://www.mdpi.com/article/10.3390/ijms26083791/s1.

## Abbreviations

The following abbreviations are used in this manuscript:

bAVM	Brain arteriovenous malformations
ECM	Extracellular matrix
YAP	Yes-associated protein
TAZ	Transcription regulator 1
CTGF	connective tissue growth factor
CYR61	cysteine-rich angiogenic inducer 61
VEGF	vascular endothelial growth factor
HIF-1α	Hypoxia-inducible factor 1-α
FFPE	Formalin-fixed paraffin-embedded
H&E	Hematoxylin–eosin
