# Supplementary figures and images for: The Role of Hippo Signaling in Brain Arteriovenous Malformations: Molecular Insights into Post-Embolization Remodeling
Source: Int J Mol Sci. 2025 Apr 17;26(8):3791. doi: 10.3390/ijms26083791 (PMC12028238; doi:10.3390/ijms26083791)

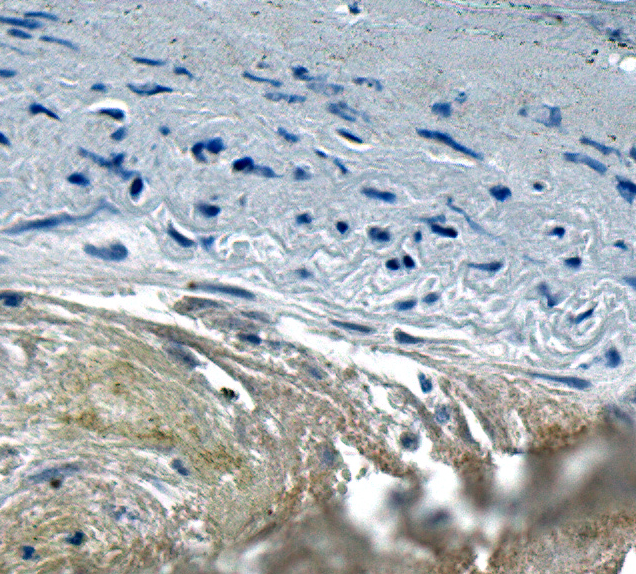

Supplement: Supplementary file 1 [file ijms-26-03791-s001.zip › Supplemental/CTGF_ZOOM_bAVM.tif]

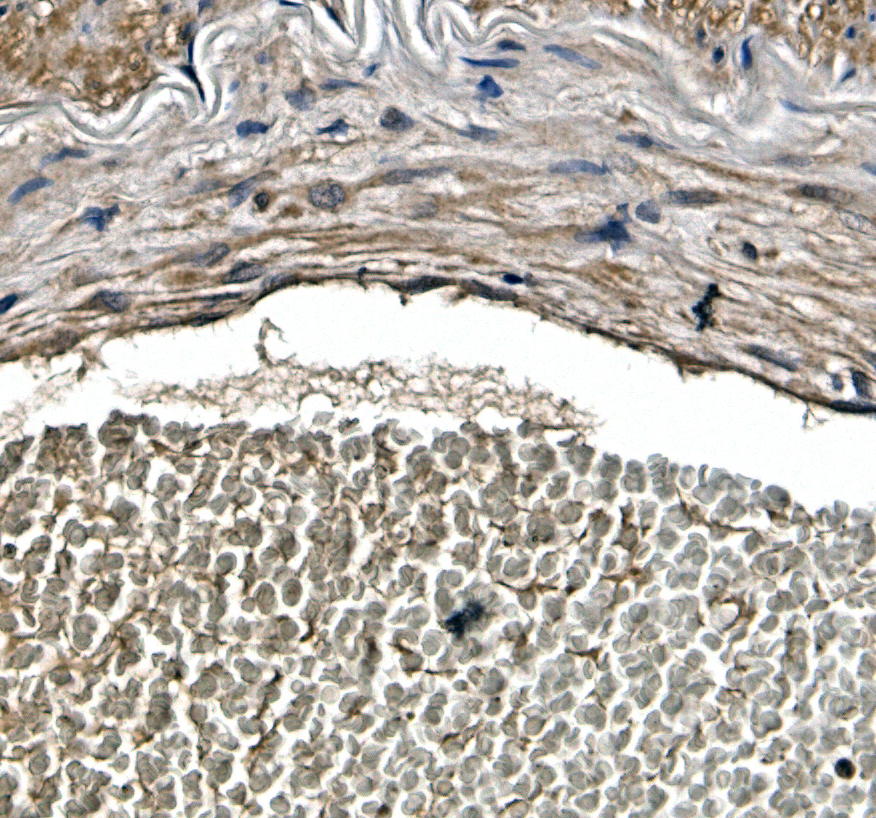

Supplement: Supplementary file 1 [file ijms-26-03791-s001.zip › Supplemental/CTGF_ZOOM_Control.tif]

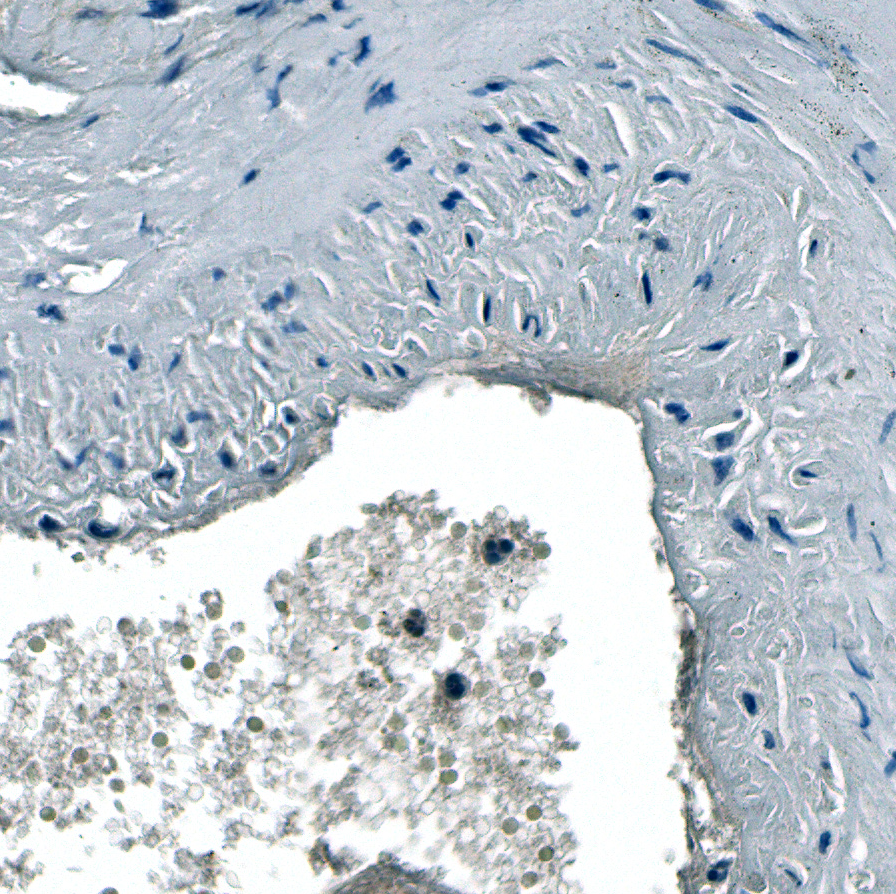

Supplement: Supplementary file 1 [file ijms-26-03791-s001.zip › Supplemental/CYR_ZOOM_bAVM.tif]

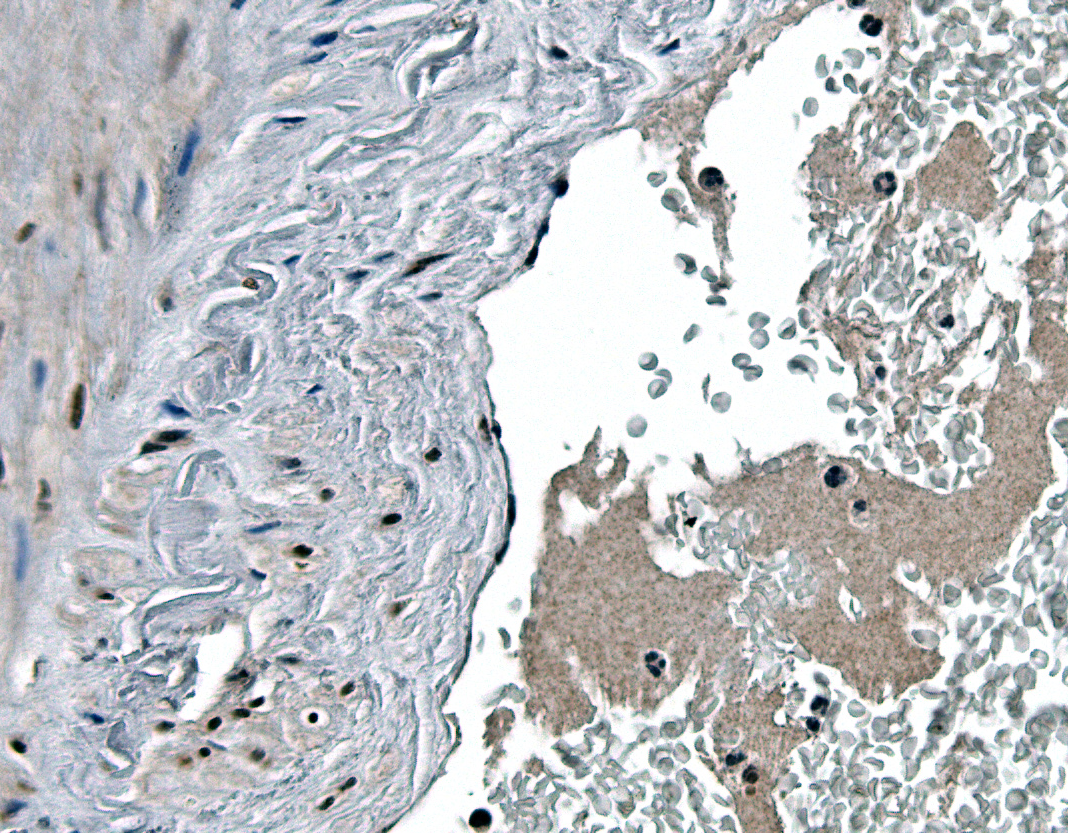

Supplement: Supplementary file 1 [file ijms-26-03791-s001.zip › Supplemental/CYR_ZOOM_Control.tif]

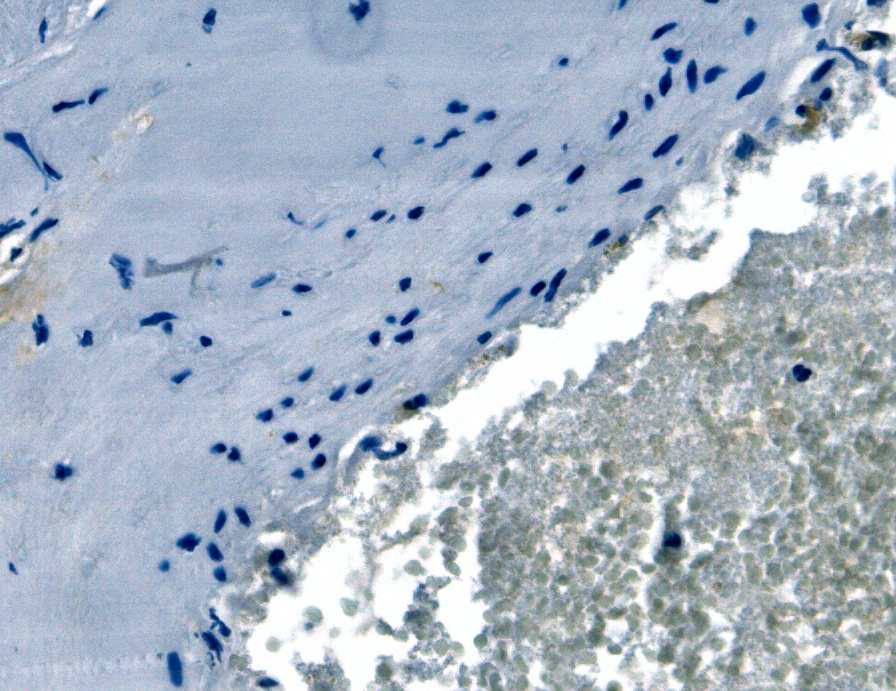

Supplement: Supplementary file 1 [file ijms-26-03791-s001.zip › Supplemental/YAP_ZOOM_BAVM.tif]

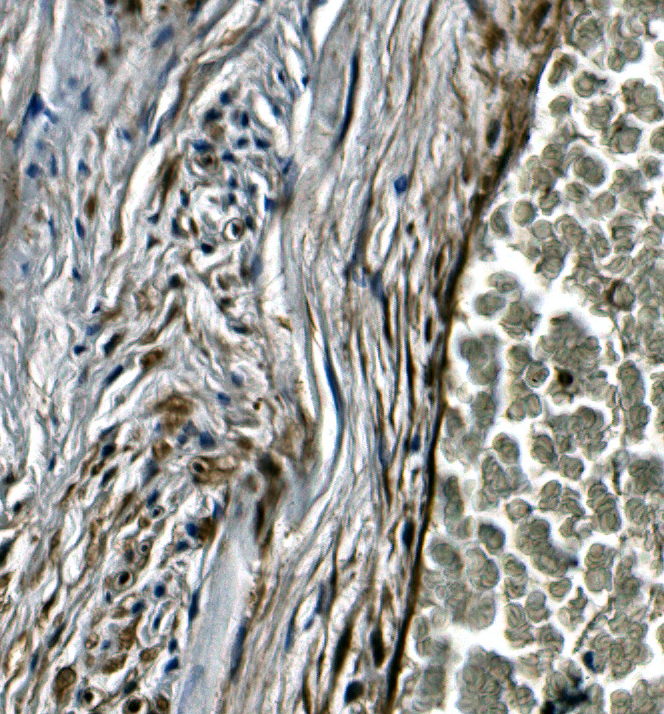

Supplement: Supplementary file 1 [file ijms-26-03791-s001.zip › Supplemental/YAP_ZOOM_Control.tif]
